# Supplementary material for: A Standardised Core Outcome Set for Measurement and Reporting Sedentary Behaviour Interventional Research: The CROSBI Consensus Study
Source: Int J Environ Res Public Health. 2022 Aug 5;19(15):9666. doi: 10.3390/ijerph19159666 (PMC9367894; doi:10.3390/ijerph19159666)
Supplement: Supplementary file 1 [file ijerph-19-09666-s001.zip › ijerph-1831172-supplementary.pdf]

Supplementary Figure S1. Flow Diagram of Systematic Review.

PRISMA 2009 Flow Diagram

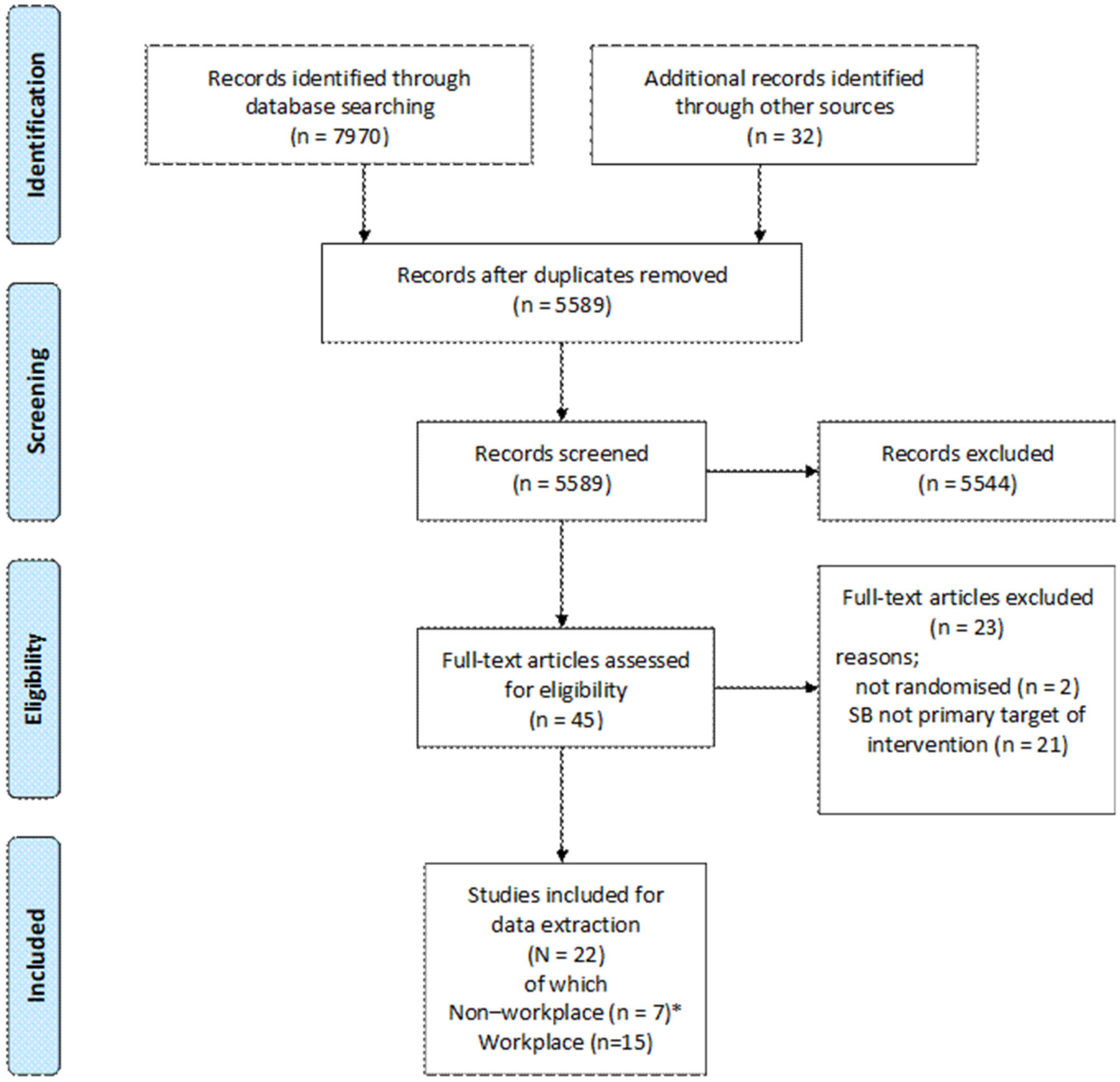

* Curran F, Blake C, Cunningham C, Perrotta C, van der Ploeg H, Matthews J, et al. (2021) Efficacy, characteristics, behavioural models and behaviour change strategies, of non-workplace interventions specifically targeting sedentary behaviour; a systematic review and meta-analysis of randomised control trials in healthy ambulatory adults. PLoS ONE 16(9): e0256828. https://doi.org/10.1371/journal.pone.0256828

From Moher D, Liberati A, Tetzlaff J, Altman DG; PRISMA Group. Preferred reporting items for systematic reviews and meta-analyses: the PRISMA statement. PLoS Med. 2009 Jul 21;6(7):e1000097. doi: 10.1371/journal.pmed.1000097. Epub 2009 Jul 21. PMID: 19621072; PMCID: PMC2707599.

Supplementary Table S1. Data Domains and Data Items Identified for Initial Expert Panel Review.

**Domain**	**Item**
Demographic (9)	Age
	Gender
	Employment Status
	Occupation
	Education level
	Ethnicity
	Health status
measure at	At baseline
	At follow-up
Device Type (5)	Inclinometer
	Accelerometer
	Combined Inclinometer/Accelerometer
	Pedometer
	Other
Device Validity (3)	Minimum wear time hours/ day
	Minimum wear time days /week
	Device position
Device wear-time (3)	Total daily wear-time
	Waking non/sleep wear-time
	Sleep wear-time
Sedentary pattern outcomes (10)	Sitting time
	Standing time
	Stepping time
	Sedentary breaks
	Short sedentary bouts
	Prolonged sedentary bouts

	Standing bouts
	Stepping bouts
measure	At baseline
	At follow-up
Reporting Measures of Sitting, Standing, Stepping time (13)	mins/day
	hours/day
	mins/workday
	hours/workday
	mins/non-work day
	hours/non-workday
	mins/work hours on workday
	mins/non-work hours on workday
	% daily waking hours
	% of work hours
	% of total daily hours
	% time of non-work hours
	mins/8 hour leisure
Reporting Measures of Sedentary breaks (5)	n/day
	n/work day
	n/non-work day
	n/hours of workplace sitting
	n/non work time on workday
Reporting Measures of Sedentary bouts (14)	prolonged sitting bout measured as<br>A. >30min
	B. >35min
	C. >60min
	short sitting bout measured as<br>A. <10min

	B. <20min
	C. <30min
	D. <35min
	longest sitting bout mins
	average duration sitting bout mins
	total duration sitting bouts mins/day
	n/day
	n/work day
	n/non-work day
	n/hours of workplace sitting
	n/non-work time on workday
	mins/day
	hours/day
	mins/workday
	n/hour workday
Standing bouts (1)	n/day
Stepping bouts (1)	n/day
Statistic to report for all core Sedentary Pattern outcomes (2)	Mean and Standard deviation Pre and Post -intervention (baseline and follow- up)
	Change in mean and CI for each outcome
Measures of physical activity (5)	Light intensity physical activity
	Moderate intensity physical activity
	Vigorous intensity physical activity
	Moderate-Vigorous intensity physical activity
	Physical Activity Energy Expenditure (kj/kg/day)
Reporting of time in each Physical activity (6)	mins/day
	min/8hour leisure
	mins/workday
	% of daily waking hours

	% wear-time on work days
	% wear-time during work hours
Reporting Physical Activity Energy Expenditure (kj/kg/day) (4)	Waking hours, all days
	Waking hours, working day
	Waking hours, non-working days
	Working hours, working days
Statistic to report for all core physical activity outcomes (2)	Mean and Standard deviation Pre and Post -intervention (baseline and follow- up)
	Change in mean and CI for each outcome
Anthropometric Measures (6)	Weight (kg)
	Height (cm)
	BMI (body mass index)
	Waist (cm^2)
	Body Fat (%)
	Fat free mass (%)
Statistic to report for all core Anthropometric outcomes (2)	Mean and Standard deviation Pre and Post -intervention (baseline and follow- up)
	Change in mean and CI for each outcome
Biomarkers (17)	Fasting Plasma Glucose (mmol/L)
	Fasting Serum insulin (pmol/L)
	HbA1c (%)
	HOMA-IR
	HOMA-%B
	Total Cholesterol (mmol/L)
	HDL Cholesterol (mmol/L)
	LDL Cholesterol (mmol/L)
	Non LDL Cholestrol (mmol/L)
	LDL/HDL Cholestrol

	Triglyerides (mmol/L)
	Aortic Augmentation Index (%)
	Subendocardial Variability (%)
	Systolic BP (mmHg)
	Diastolic BP (mmHg)
	Estimated VO2 (mL/kg/min)
	Resting Heart Rate
Statistic to report for all core biometric outcomes (2)	Mean and Standard deviation Pre and Post -intervention (baseline and follow- up)
	Change in mean and CI for each outcome
Domains = 19	Domain Items = 110

Mins, minutes; n/, number per; CI, confidence interval; kj/kg/day, kilojoules per kilogram per day; kg, kilogram; cm, centimetre; mmol/l, millimole per litre; pmol/l, picomoles per litre; HbA1c,; HOMA-IR, Homeostatic Model Assessment for Insulin Resistance; HOMA-%B, homeostatic model assessment to quantify beta-cell function; HDL, high density lipoprotein; LDL, low density lipoprotein; BP (mmHg), Blood Pressure in millimetres of mercury; VO2 (mL/kg/min), Volume of oxygen in millilitre per kilogram per minute.

Supplementary Table S2. Additional Items identified by Expert Review.

Additional items for inclusion from expert panel review	
Domain	Item
Demographics	Population type (Healthy /clinical)
Device Type	smart watch
Device Type	Make
Device Type	Model
Device Type	Manufacturer
Sedentary pattern outcomes	Sedentary time
Sedentary pattern outcomes	Sleeping Time /LPA/ MVPA separately
Sedentary breaks	number of steps /day

LPA, light physical activity; MVPA, moderate to vigorous physical activity;

Supplementary Table S3. Data Items for round 1 Delphi.

Outcome code	Item	HelpText
**01.01**	Age	How old participants are/the age (in years) of participants
**01.02**	Gender	How participants identify themselves with regard to being male, female, or non-binary/third gender
**01.03**	Employment Status	Whether participants are in paid employment, voluntary employment, unemployed, retired, student in education, other
**01.04**	Occupation	Whether participants employment is active, semi-active, sedentary
**01.05**	Education level	Highest education level achieved by participant (Primary /Secondary/ Tertiary)
**01.06**	Ethnicity	The social group with common national and cultural tradition that participants identify as belonging to, e.g., white/Caucasian, Asian, black/African/Caribbean/other
**01.07**	Population type	Whether participants are healthy sedentary, healthy active or a clinical cohort
**01.08**	Weight	The amount that a participant weighs (measured in Kilos or pounds)
**01.09**	Height	Height of a participant while standing on a hard surface (measured in centimetres or inches)
**01.10**	BMI (body mass index)	An approximate measure of whether a participant is overweight or underweight, calculated by dividing their weight in kilograms by the square of their height in metres
**01.11**	Measured at baseline	Population measures at baseline (pre-intervention)

**01.12**	Measured at follow-up	Population measures at Follow-up (Post intervention))
**01.13**	Report as; Mean, standard deviation, and range, or number or % in each category, across all for participants at baseline	numerical population measures reported as mean, SD and range e.g. weight; categorical measures reported as number or % in each e.g., Ethnicity
**01.14**	Report as; Mean, standard deviation, and range, or number or % in each category, across all for participants at follow-up	numerical population measures reported as mean, SD and range e.g., weight; categorical measures reported as number or % in each e.g., Ethnicity
**02.01**	Inclinometer	a device which captures differences in body position (e.g., sitting vs. lying vs. standing)
**02.02**	Accelerometer	an activity tracking device which captures acceleration of movement and categorises it into different intensities e.g., ActiGraph GT3X accelerometer
**02.03**	Combined Inclinometer/Accelerometer	a device which captures information about acceleration and also body position (i.e., sitting/lying, standing and stepping) e.g., activPAL
**02.04**	Pedometer	a device which records the number of steps taken (e.g., jawbone ; fitbit;)
**02.05**	Smartwatch	a wrist-worn device with activity or fitness tracking capability, connected to mobile technology
**02.06**	Other	please specify if other type of device
**03.01**	Device type	the name of make, model and manufacturer of the device used to record outcomes
**03.02**	Device sensor position	the position where the device sensors are worn or attached to the participants body
**03.03**	Device minimum wear time hours / day	the minimum number of hours per day that participants must wear the device to be included in the analysis
**03.04**	Device minimum wear time days /week	the minimum number of days that participants must wear the device to be included in the analysis

**04.01**	Device; total daily wear-time	the total amount of time, in hours or minutes, that the device is worn over 24 hours
**04.02**	Device; waking / non-sleep wear-time	the amount of time, in hours or minutes per day, that the device is worn while the participant is awake / not sleeping
**04.03**	Device; Sleep wear-time	the amount of time, in hours or minutes per day, that the device is worn while the participant is sleeping
**04.04**	Report as; mean, standard deviation and range,	mean, standard deviation and range, across all participants for measures of device wear time
**05.01**	Sedentary time	time, in hours or minutes, that participant spent in sitting, reclining or lying, while awake.
**05.02**	Sitting time	time, in hours or minutes, that participant spent in sitting
**05.03**	Standing time	time, in hours or minutes, that participant spent in standing
**05.04**	Stepping time	time, in hours or minutes, that participant spent in stepping
**05.05**	Measure at baseline	Posture measures at baseline (pre-intervention)
**05.06**	Measure at follow-up	Posture measures at follow-up (post-intervention)
**06.01**	Daily time, in the specified posture	time, in hours or minutes per day, that participant spent in the specified posture i.e. in sedentary time; sitting time; standing time; stepping time
**06.02**	Work day, daily time in the specified posture	time, in hours or minutes **per workday**, that participant spent in sitting, reclining or lying while awake
**06.03**	Non-Work day, in the specified posture	time, in hours or minutes **per non-workday**, that participant spent in the specified posture i.e. in sedentary time; sitting time; standing time; stepping time
**06.04**	hourly time during work hours, on a workday, in the specified posture	time in minutes **per hour at work on a workday**, that participant spent in the specified posture i.e. in sedentary time; sitting time; standing time; stepping time

**06.05**	hourly time during NON-work hours on a workday, in the specified posture	time in minutes **per hour while not at work on a workday**, that participant spent in the specified posture i.e. in sedentary time; sitting time; standing time; stepping time
**06.06**	% daily waking hours	% of daily waking hours that participant spent in the specified posture i.e. in sedentary time; sitting time; standing time; stepping time
**06.07**	% of work hours	% of work hours that participant spent in the specified posture i.e., in sedentary time; sitting time; standing time; stepping time
**06.08**	% of total daily hours	% of total daily hours that participant spent in the specified posture i.e. in sedentary time; sitting time; standing time; stepping time
**06.09**	% time of non-work hours	% time of non-work hours that participant spent in the specified posture i.e. in sedentary time; sitting time; standing time; stepping time
**06.10**	Report as; Mean, standard deviation, and range for participants, Pre and Post -intervention (baseline and follow- up)	report the mean, standard deviation, and range, across all participants, Pre and Post -intervention (baseline and follow-up)
**06.11**	Report as; change in mean, and confidence interval for each outcome	report the change in mean, and the confidence interval for postural time measures across all participants
**07.01**	Sedentary breaks; Sit to stand or upright transitions	A non-sedentary bout between two sedentary bouts (measured as a change in posture from sitting, reclining or lying to standing posture)
**07.02**	Sedentary breaks; Movement breaks	A non-sedentary, non-stationary bout between two sedentary bouts i.e. includes moving e.g. walking
**07.03**	Sedentary breaks; Number of steps	The number of steps the participant takes
**07.04**	Measure at baseline	Sedentary breaks, measured at baseline (pre-intervention)
**07.05**	Measure at follow-up	Sedentary breaks measured at follow-up (post-intervention)

**08.01**	n/day ; number of sedentary breaks daily	number of sedentary breaks by the participant per day; for use with core outcome, sit-stand transitions / upright movements, and / or, core outcome, movement breaks
**08.02**	n/work day; number of sedentary breaks per work day	number of sedentary breaks by the participant per WORK day; for use with core outcome, sit-stand transitions / upright movements, and /or, core outcome, movement breaks
**08.03**	n/non-work day; number of sedentary breaks per non work day	number of sedentary breaks by the participant per NON-work day / leisure day; for use with core outcome, sit-stand transitions / upright movements, and / or, core outcome, movement breaks
**08.04**	n/hours of workplace sitting; of sedentary breaks	number of sedentary breaks by participant per hours of sitting at work; for use with core outcome, sit-stand transitions / upright movements, and / or, core outcome, movement breaks
**08.05**	n/non work time on workday; of sedentary breaks	number of sedentary breaks by the participant while not at work / during leisure time on a work day; for use with core outcome, sit-stand transitions / upright movements, and / or, core outcome, movement breaks
**08.06**	Report as; mean, standard deviation, and range for participants, Pre and Post -intervention (baseline and follow- up)	report mean, standard deviation, and range pre and post -intervention (baseline and follow- up), for measures of sedentary breaks, across all participants
**08.07**	Report as; change in mean, and confidence interval, or change in rate, for each outcome	report the change in mean, and the confidence interval for measures of sedentary breaks across all participants
**09.01**	Short sedentary bouts <10 minutes	A short period of uninterrupted sitting, reclining or lying time of less than 10 minutes
**09.02**	Prolonged sedentary bouts >30 minutes	A prolonged period of uninterrupted sitting, reclining or lying time of more than 30 minutes
**09.03**	Prolonged sedentary bouts >60 minutes	A prolonged period of uninterrupted sitting, reclining or lying time of more than 60 minutes
**09.04**	Standing bouts	A period of uninterrupted standing time

**09.05**	Stepping bouts	A period of uninterrupted stepping time
**09.06**	Measure at baseline	Sedentary bouts measured at baseline (pre-intervention)
**09.07**	Measure at follow-up	Sedentary bouts measured at follow-up (post-intervention)
**10.01**	average duration of bout, mins	average duration of participants bout in mins
**10.02**	total duration of bouts, mins or hours /day	total duration in minutes or hours per day, of time accumulated in specified bouts
**10.03**	n/day	number of specified bouts per day
**10.04**	n/work day	number of specified bouts per work day
**10.05**	n/non-work day	number of specified bouts per NON work day
**10.06**	n/hours of workplace sitting	number of specified bouts per hours sitting at work
**10.07**	n/non-work time on workday	number of specified bouts per non-work time on workday
**10.08**	total duration of bouts, mins or hours / workday	time in minutes or hours per WORK day, accumulated in specified bouts
**10.09**	Report as; mean, standard deviation, and range for participants, Pre and Post -intervention (baseline and follow- up)	report mean, standard deviation, and range pre and post -intervention (baseline and follow- up), for measures of sedentary bouts, across all participants
**10.10**	Report as; change in mean, and confidence interval, or change in rate for each outcome	report the change in mean, and the confidence interval for measures of sedentary bouts across all participants
**11.01**	Light intensity physical activity, time	activity performed at under 3 METs, including casual walking, doing household shores or activities of daily living
**11.02**	Moderate intensity physical activity, time	activity performed at between 3 and 6 METs, e.g., brisk walking, cycling, dancing. Double’s tennis.

**11.03**	Vigorous intensity physical activity, time	activity performed ≥6 METs, e.g., hiking uphill, running, swimming laps, aerobic dancing, tennis.
**11.04**	Moderate-vigorous intensity physical activity, time	activity performed at ≥3 and including some ≥ 6 METs
**11.05**	Physical Activity Energy Expenditure (kj/kg/day)	The amount of energy used by participant per kilo of body weight per day
**11.06**	Measure at baseline	Physical activity measures at baseline (pre-intervention)
**11.07**	Measure at follow-up	Physical activity measures at follow-up (post-intervention)
**12.01**	mins/day	Time in minutes per day, spent by participant in the specified PA (light; moderate; vigorous; moderate-vigorous)
**12.02**	mins/workday	Time, in minutes per work day, spent by participant in the specified PA (light; moderate; vigorous; moderate-vigorous)
**12.03**	% of daily waking hours	Time as a % of daily waking hours, spent by participant in the specified PA (light; moderate; vigorous; moderate-vigorous)
**12.04**	% wear time on work days	Time as % of wear time on work days, spent by participant in the specified PA (light; moderate; vigorous; moderate-vigorous)
**12.05**	% wear time during work hours	Time as % of wear time during work hours, spent by participant in the specified PA (light; moderate; vigorous; moderate-vigorous)
**12.06**	Report as; mean, standard deviation, and range for participants, Pre and Post -intervention (baseline and follow- up)	report mean, standard deviation, and range pre and post -intervention (baseline and follow- up), for measures of physical activity energy expenditure, across all participants
**12.07**	Report as; change in mean, and confidence interval, for each outcome	report the change in mean, and the confidence interval for measures of physical activity across all participants
**13.01**	Waking hours, all days	Energy expenditure while awake for all days (work days and non-work days)

**13.02**	Waking hours, working day	Energy expenditure while awake on work days
**13.03**	Waking hours, non-working days	Energy expenditure while awake for on non-work days
**13.04**	Working hours, working days	Energy expenditure while at during working hours on workdays
**13.05**	Report as; mean, standard deviation, and range for participants, Pre and Post -intervention (baseline and follow- up)	report mean, standard deviation, and range pre and post - intervention (baseline and follow- up), for measures of physical activity, across all participants
**13.06**	Report as; change in mean, and confidence interval, for each outcome	report the change in mean, and the confidence interval for measures of physical activity energy expenditure, across all participants

SD, standard deviation; n/, number per; mins, minutes; METs metabolic equivalents; kj/kg/day, kilojoule per kilogram per day; PA, physical activity;

Supplementary Table S4. Round 1 Delphi Suggested Additional Items.

**Round 1 suggested additional items**
Urban versus rural dwelling
Ability level (people living with a disability; chronic condition; special needs)
Sedentary time pattern outcomes; half bout duration; fragmentation of sedentary bouts; variation of bout length
baseline compositional values
bout median
half-life bout duration
Medications use that is likely to interact with the intervention e.g. metformin
Social economic status
Presence of chronic disease

Domain or behaviour specific behaviours (via observation or self-report)
Statistical outcomes should be reported using median and IQR if distributions are not normal.
All data should be reported per 24 hours if at all possible.
Self-reported sitting time (to see if perceptions differ from device measures)
Urban versus rural dwelling

IQR, Inter Quartile Range.

Supplementary Table S5. Delphi Round 1 Ratings.

Item		Round 1 rating					
	1-3 (≤15%)	4-6	7-9 (≥50%)	median	n	Include in round 2 and reason	
Age	2%	2%	96%	9	50	no	to reduce burden
Gender	2%	8%	90%	9	49	no	to reduce burden
Employment Status	2%	44%	54%	7	50	yes	meets in criteria
Occupation	2%	48%	50%	6.5	50	yes	meets in criteria
Education level	4%	50%	46%	6	50	no	meets out criteria
Ethnicity	10%	44%	46%	6	50	no	meets out criteria
Population type	2%	30%	68%	7	47	yes	meets in criteria

Weight	12%	30%	58%	7	50	yes	meets in criteria
Height	14%	34%	52%	7	50	yes	meets in criteria
BMI (body mass index)	4%	33%	63%	7	49	yes	meets in criteria
Measured at baseline	0%	6%	94%	9	50	no	to reduce burden
Measured at follow-up	6%	14%	80%	9	50	no	to reduce burden
Report as; Mean, standard deviation, and range, or number or % in each category, across all for participants at baseline	0%	6%	94%	9	48	no	to reduce burden
Report as; Mean, standard deviation, and range, or number or % in each category, across all for participants at follow-up	0%	15%	85%	9	47	no	to reduce burden
Standardised clinical / health related measures							Additional outcome
Inclinometer	5%	12%	84%	8	43		meets in criteria
Accelerometer	7%	26%	67%	7	46		meets in criteria
Combined Inclinometer/Accelerometer	7%	16%	78%	9	45		meets in criteria
Pedometer	67%	27%	6%	3	48	no	exclusion criteria met
Smartwatch	33%	48%	19%	5	48	no	exclusion criteria met
Other	44%	24%	32%	5	25	no	exclusion criteria met

Device type (Make / Model /Manufacturer)	0%	2%	98%	9	49	no	to reduce burden
Device sensor position	0%	2%	98%	9	49	no	to reduce burden
Device minimum wear time hours / day	2%	0%	98%	9	50	no	to reduce burden
Device minimum wear time days /week	2%	6%	92%	9	50	no	to reduce burden
Device; total daily wear-time	4%	4%	92%	9	50	yes	meets in criteria
Device; waking / non-sleep wear-time	2%	20%	78%	9	50	yes	meets in criteria
Device; Sleep wear-time	6%	26%	68%	8	50	yes	meets in criteria
Report as; mean, standard deviation and range,	0%	6%	94%	9	49	no	to reduce burden
Sedentary time	2%	6%	92%	9	50	yes	meets in criteria
Sitting time	2%	10%	88%	9	50	yes	meets in criteria
Standing time	4%	16%	80%	9	50	yes	meets in criteria
Stepping time	8%	18%	74%	8	50	yes	meets in criteria
Measure at baseline	0%	0%	100%	9	49	no	to reduce burden
Measure at follow-up	0%	0%	100%	9	49	no	to reduce burden
Standardised self-report or observational measures including domain or behavior specific behaviours							Additional outcome

Daily time, in the specified posture	2%	8%	90%	9	50	yes	meets in criteria
Work day, daily time in the specified posture	10%	42%	48%	6	50	yes	out criteria not met
Non-Work day, in the specified posture	10%	44%	46%	6	50	yes	out criteria not met
hourly time during work hours, on a workday, in the specified posture	12%	46%	42%	6	50	yes	out criteria not met
hourly time during NON-work hours on a workday, in the specified posture	12%	50%	38%	6	50	no	exclusion criteria met
% daily waking hours	6%	22%	72%	8	50	yes	meets in criteria
% of work hours	14%	30%	56%	7	50	yes	meets in criteria
% of total daily hours	14%	12%	74%	8	50	yes	meets in criteria
% time of non-work hours	16%	32%	52%	7	50	yes	meets in criteria
Report as; mean, standard deviation, and range for participants, Pre and Post -intervention (baseline and follow-up)	0%	6%	94%	9	49	no	to reduce burden
Report as; change in mean, and confidence interval for each outcome	2%	19%	79%	8.5	48	no	to reduce burden
Sedentary breaks; Sit to stand or upright transitions	4%	26%	70%	7.5	50	yes	meets in criteria
Sedentary breaks; Movement breaks	10%	18%	72%	7	50	yes	meets in criteria
Sedentary breaks; Number of steps	10%	43%	47%	6	49	yes	meets in criteria

Measure at baseline	4%	10%	86%	9	50	no	to reduce burden
Measure at follow-up	4%	10%	86%	9	50	no	to reduce burden
n/day ; number of sedentary breaks daily	4%	26%	70%	7	50	yes	meets in criteria
n/work day; number of sedentary breaks per work day	14%	46%	40%	6	50	yes	out criteria not met
n/non-work day; number of sedentary breaks per non work day	14%	46%	40%	6	50	yes	out criteria not met
n/hours of workplace sitting; of sedentary breaks	13%	35%	52%	7	48	yes	meets in criteria
n/non work time on workday; of sedentary breaks	12%	43%	45%	6	49	yes	out criteria not met
Report as; mean, standard deviation, and range for participants, Pre and Post -intervention (baseline and follow-up)	2%	15%	83%	8	48	no	to reduce burden
Report as; change in mean, and confidence interval, or change in rate, for each outcome	4%	25%	71%	8	48	no	to reduce burden
Short sedentary bouts <10 minutes	16%	42%	42%	6	50	no	meets in criteria
Prolonged sedentary bouts >30 minutes	2%	22%	76%	8	50	yes	meets in criteria
Prolonged sedentary bouts >60 minutes	8%	18%	74%	8	50	yes	meets in criteria
Standing bouts	12%	42%	46%	6	50	yes	out criteria not met
Stepping bouts	12%	38%	50%	6.5	50	yes	meets in criteria
Measure at baseline	0%	14%	86%	9	50	no	to reduce burden

Measure at follow-up	0%	14%	86%	9	50	no	to reduce burden
average duration of bout, mins	6%	30%	64%	7	50	yes	meets in criteria
total duration of bouts, mins or hours /day	8%	28%	64%	7	50	yes	meets in criteria
n/day	12%	36%	52%	7	50	yes	meets in criteria
n/work day	20%	46%	34%	6	50	no	exclusion criteria met
n/non-work day	24%	42%	34%	6	50	no	exclusion criteria met
n/hours of workplace sitting	14%	39%	47%	6	49	yes	out criteria not met
n/non-work time on workday	20%	36%	44%	6	50	no	exclusion criteria met
total duration of bouts, mins or hours / workday	16%	40%	44%	6	50	no	exclusion criteria met
Report as; mean, standard deviation, and range for participants, Pre and Post -intervention (baseline and follow-up)	2%	15%	83%	8	48	no	to reduce burden
Report as; change in mean, and confidence interval, or change in rate for each outcome	4%	21%	75%	7	48	no	to reduce burden
Weighted median bout duration or bout "half-life"							Additional outcome
Light intensity physical activity, time	0%	20%	80%	9	50	yes	meets in criteria

Moderate intensity physical activity, time	0%	22%	78%	9	50	yes	meets in criteria
Vigorous intensity physical activity, time	0%	24%	76%	8.5	50	yes	meets in criteria
Moderate-Vigorous intensity physical activity, time	0%	24%	76%	8.5	50	yes	meets in criteria
Physical Activity Energy Expenditure (kj/kg/day)	22%	49%	29%	5	49	no	exclusion criteria met
Measure at baseline	0%	16%	84%	9	50	no	to reduce burden
Measure at follow-up	0%	14%	86%	9	50	no	to reduce burden
mins/day	4%	4%	92%	9	49	yes	meets in criteria
mins/workday	13%	42%	46%	6	48	no	exclusion criteria met
% of daily waking hours	15%	19%	67%	7.5	48	no	out criteria not met
% wear-time on work days	21%	35%	44%	6	48	no	exclusion criteria met
% wear-time during work hours	21%	36%	43%	6	47	no	exclusion criteria met
Report as; mean, standard deviation, and range for participants, Pre and Post -intervention (baseline and follow-up)	2%	10%	88%	9	49	no	to reduce burden
Report as; change in mean, and confidence interval, for each outcome	6%	18%	76%	8	49	no	to reduce burden

Waking hrs, all days	16%	34%	50%	6.5	50	no	Parent domain excluded
Waking hours, working day	16%	60%	24%	5	50	no	Parent domain excluded
Waking hrs, non-working days	16%	60%	24%	5	50	no	Parent domain excluded
Working hrs, working days	18%	62%	20%	5	50	no	Parent domain excluded
Report as; mean, standard deviation, and range for participants, Pre and Post -intervention (baseline and follow-up)	12%	31%	57%	8	49	no	Parent domain excluded
Report as; change in mean, and confidence interval, for each outcome	18%	31%	51%	7	49	no	Parent domain excluded

Supplementary Table S6. Delphi Round 2 Ratings.

**Item**	**Round 2 rating**						
	**1-3 (≤15%)**	**4-6**	**7-9 (≥70%)**	**median**	**n**		**Include in COS and reason**
Age	0%	0%	100%	9	41	yes	meets in criteria R1 & R2
Gender	0%	7%	93%	9	41	yes	meets in criteria R1 & R2

Employment Status	2%	37%	61%	7	41	no	R2 Include criteria not met
Occupation	0%	46%	54%	7	41	no	R2 Include criteria not met
Education level	5%	51%	44%	6	41	no	R2 Include criteria not met
Ethnicity	10%	46%	44%	6	41	no	R2 Include criteria not met
Population type	3%	26%	72%	7	39	yes	R2 meets include criteria
Weight	12%	27%	61%	7	41	no	R2 Include criteria not met
Height	17%	24%	59%	7	41	no	R2 Include criteria not met
BMI (body mass index)	3%	25%	73%	9	40	yes	meets in criteria R1 & R2
Measured at baseline						yes	meets in criteria R1 (Obvious)
Measured at follow-up						yes	meets in criteria R1 (Obvious)
Report as; mean, standard deviation, and range, or number or % in each category, across all for participants at baseline						yes	meets in criteria R1 (Obvious)
Report as; Mean, standard deviation, and range, or number or % in each category, across all for participants at follow-up						yes	meets in criteria R1 (Obvious)
Standardised clinical / health related measures	12%	39%	48%	6	33	no	R2 Include criteria not met
Inclinometer	3%	13%	85%	9	39	yes	meets in criteria R1 & R2

Accelerometer	5%	28%	68%	7	40	no	R2 Include criteria not met
Combined Inclinometer/Accelerometer	3%	13%	84%	9	38	yes	meets in criteria R1 & R2
Pedometer						no	R1 exclusion criteria met
Smartwatch						no	R1 exclusion criteria met
Other						no	R1 exclusion criteria met
Device type (Make / Model /Manufacturer)						yes	meets in criteria R1 (Obvious)
Device sensor position						yes	meets in criteria R1 (Obvious)
Device minimum wear time hours / day						yes	meets in criteria R1 (Obvious)
Device minimum wear time days /week						yes	meets in criteria R1 (Obvious)
Device; total daily wear-time	5%	0%	95%	9	41	yes	meets in criteria R1 & R2
Device; waking / non-sleep wear-time	2%	2%	95%	9	41	yes	meets in criteria R1 & R2
Device; Sleep wear-time	2%	7%	90%	9	41	yes	meets in criteria R1 & R2
Report as; mean, standard deviation and range,						yes	meets in criteria R1 (Obvious)
Sedentary time	2%	2%	95%	9	41	yes	meets in criteria R1 & R2
Sitting time	2%	7%	90%	9	41	yes	meets in criteria R1 & R2

Standing time	2%	7%	90%	9	41	yes	meets in criteria R1 & R2
Stepping time	7%	10%	83%	9	41	yes	meets in criteria R1 & R2
Measure at baseline						yes	meets in criteria R1 (Obvious)
Measure at follow-up						yes	meets in criteria R1 (Obvious)
Standardised self-report or observational measures including domain or behaviour specific behaviours	6%	31%	63%	7	35	no	R2 Include criteria not met
Daily time, in the specified posture	2%	5%	93%	9	41	yes	meets in criteria R1 & R2
Work day, daily time in the specified posture	10%	63%	27%	6	41	no	R2 Include criteria not met
Non-Work day, in the specified posture	10%	63%	27%	6	41	no	R2 Include criteria not met
hourly time during work hours, on a workday, in the specified posture	17%	61%	22%	6	41	no	R2 Include criteria not met
hourly time during NON-work hours on a workday, in the specified posture	17%	61%	22%	6	41	no	R2 Include criteria not met
% daily waking hours	5%	17%	78%	9	41	yes	meets in criteria R1 & R2
% of work hours	5%	46%	49%	6	41	no	R2 Include criteria not met
% of total daily hours	10%	10%	80%	8	41	yes	meets in criteria R1 & R2
% time of non-work hours	10%	39%	51%	7	41	no	R2 Include criteria not met

Report as; Mean, standard deviation, and range for participants, Pre and Post - intervention (baseline and follow- up)						yes	meets in criteria R1 & R2
Report as; change in mean, and confidence interval for each outcome						yes	meets in criteria R1 & R2
Sedentary breaks; Sit to stand or upright transitions	5%	17%	78%	9	41	yes	meets in criteria R1 & R2
Sedentary breaks; Movement breaks	10%	12%	78%	8	41	yes	meets in criteria R1 & R2
Sedentary breaks; Number of steps	8%	53%	40%	6	40	no	R2 Include criteria not met
Measure at baseline						yes	meets in criteria R1 (Obvious)
Measure at follow-up						yes	meets in criteria R1 (Obvious)
n/day ; number of sedentary breaks daily	10%	12%	78%	9	41	yes	meets in criteria R1 & R2
n/work day; number of sedentary breaks per work day	7%	63%	29%	6	41	no	R2 Include criteria not met
n/non-work day; number of sedentary breaks per non work day	7%	61%	32%	6	41	no	R2 Include criteria not met
n/hours of workplace sitting; of sedentary breaks	12%	44%	44%	6	41	no	R2 Include criteria not met
n/non work time on workday; of sedentary breaks	12%	51%	37%	6	41	no	R2 Include criteria not met
Report as; mean, standard deviation, and range for participants, Pre and Post - intervention (baseline and follow- up)						yes	meets in criteria R1 (Obvious)
Report as; change in mean, and confidence interval, or change in rate, for each outcome						yes	meets in criteria R1 (Obvious)

Short sedentary bouts <10 minutes						no	R1 exclusion criteria met
Prolonged sedentary bouts >30 minutes	2%	15%	83%	9	41	yes	meets in criteria R1 & R2
Prolonged sedentary bouts >60 minutes	10%	12%	78%	9	41	yes	meets in criteria R1 & R2
Standing bouts	5%	41%	54%	7	41	no	R2 Include criteria not met
Stepping bouts	7%	37%	56%	7	41	no	R2 Include criteria not met
Measure at baseline						yes	meets in criteria R1 (Obvious)
Measure at follow-up						yes	meets in criteria R1 (Obvious)
average duration of bout, mins	5%	24%	71%	7	41	yes	R2 meets include criteria
total duration of bouts, mins or hours /day	7%	22%	71%	7	41	yes	R2 meets include criteria
n/day	7%	32%	61%	7	41	no	R2 Include criteria not met
n/work day						no	R1 exclusion criteria met
n/non-work day						no	R1 exclusion criteria met
n/hours of workplace sitting	17%	41%	41%	6	41	no	R2 Include criteria not met
n/non-work time on workday						no	R1 exclusion criteria met
total duration of bouts, mins or hours / workday						no	R1 exclusion criteria met

Report as; mean, standard deviation, and range for participants, Pre and Post - intervention (baseline and follow- up)						yes	meets in criteria R1 (Obvious)
Report as; change in mean, and confidence interval, or change in rate for each outcome						yes	meets in criteria R1 (Obvious)
Weighted median bout duration or bout "half-life"	15%	47%	38%	5.5	34	no	R2 Include criteria not met
Light intensity physical activity, time	0%	7%	93%	9	41	yes	meets in criteria R1 & R2
Moderate intensity physical activity, time	0%	12%	88%	9	41	yes	meets in criteria R1 & R2
Vigorous intensity physical activity, time	0%	12%	88%	9	41	yes	meets in criteria R1 & R2
Moderate-vigorous intensity physical activity, time	0%	12%	88%	9	41	yes	meets in criteria R1 & R2
Physical Activity Energy Expenditure (kj/kg/day)						no	R1 exclusion criteria met
Measure at baseline						yes	meets in criteria R1 (Obvious)
Measure at follow-up						yes	meets in criteria R1 (Obvious)
mins/day	2%	0%	98%	9	41	yes	meets in criteria R1 & R2
mins/workday	12%	49%	39%	6	41	no	R2 Include criteria not met
% of daily waking hours	12%	12%	76%	8	41	yes	R2 meets include criteria
% wear time on work days						no	R1 exclusion criteria met
% wear time during work hours						no	R1 exclusion criteria met

Report as; mean, standard deviation, and range for participants, Pre and Post - intervention (baseline and follow- up)	yes	meets in criteria R1 (Obvious)
Report as; change in mean, and confidence interval, for each outcome	yes	meets in criteria R1 (Obvious)
Waking hrs, all days	no	Parent domain excluded
Waking hours, working day	no	Parent domain excluded
Waking hrs, non-working days	no	Parent domain excluded
Working hrs, working days	no	Parent domain excluded
Report as; mean, standard deviation, and range for participants, Pre and Post - intervention (baseline and follow- up)	no	Parent domain excluded
Report as; change in mean, and confidence interval, for each outcome	no	Parent domain excluded

Supplementary Table S7. Final Core Outcome Set.

Domain	Item
Participants / Population Demographics	Age
Participants / Population Demographics	Gender
Participants / Population Demographics	Population type
Participants / Population Demographics	BMI (body mass index)
Participants / Population Demographics	Measured at baseline
Participants / Population Demographics	Measured at follow-up
Participants / Population Demographics	Report as; Mean, standard deviation, and range, or number or % in each category, across all for participants at baseline
Participants / Population Demographics	Report as; Mean, standard deviation, and range, or number or % in each category, across all participants at follow-up
Type of device to measure sedentary behaviour	Accelerometer with Inclinometry Function*
Device details and Wear time Criteria	Device type (Make / Model /Manufacturer)
Device details and Wear time Criteria	Device sensor position
Device details and Wear time Criteria	Device minimum wear time hours/day

Device details and Wear time Criteria	Device minimum wear time days/week
Device wear time	Device; total daily wear-time
Device wear time	Device; waking / non-sleep wear-time
Device wear time	Device; Sleep wear-time
Device wear time	Report as; mean, standard deviation and range,
Posture related outcomes to measure	Sedentary time
Posture related outcomes to measure	Sitting time
Posture related outcomes to measure	Standing time
Posture related outcomes to measure	Stepping time
Posture related outcomes to measure	Measure at baseline
Posture related outcomes to measure	Measure at follow-up
Report posture related outcomes as;	Daily time, in the specified posture
Report posture related outcomes as;	% daily waking hours
Report posture related outcomes as;	% of total daily hours
Report posture related outcomes as;	Report as; Mean, standard deviation, and range for participants, Pre and Post -intervention (baseline and follow- up)

Report posture related outcomes as;	Report as; change in mean, and confidence interval for each outcome
Sedentary Breaks outcomes to measure	Sedentary breaks; Sit to stand or upright transitions
Sedentary Breaks outcomes to measure	Sedentary breaks; Movement breaks
Sedentary Breaks outcomes to measure	Measure at baseline
Sedentary Breaks outcomes to measure	Measure at follow-up
Report Sedentary Break outcomes as;	n/day ; number of sedentary breaks daily
Report Sedentary Break outcomes as;	Report as; mean, standard deviation, and range for participants, Pre and Post -intervention (baseline and follow- up)
Report Sedentary Break outcomes as;	Report as; change in mean, and confidence interval, or change in rate, for each outcome
Sedentary Bouts outcomes to measure;	Prolonged sedentary bouts >30 minutes
Sedentary Bouts outcomes to measure;	Prolonged sedentary bouts >60 minutes
Sedentary Bouts outcomes to measure;	Measure at baseline
Sedentary Bouts outcomes to measure;	Measure at follow-up
Report Sedentary Bout outcomes as;	average duration of bout, mins
Report Sedentary Bout outcomes as;	total duration of bouts, mins or hours /day

Report Sedentary Bout outcomes as;	Report as; mean, standard deviation, and range for participants, Pre and Post -intervention (baseline and follow- up)
Report Sedentary Bout outcomes as;	Report as; change in mean, and confidence interval, or change in rate for each outcome
Physical Activity outcomes to measure;	Light intensity physical activity, time
Physical Activity outcomes to measure;	Moderate intensity physical activity, time
Physical Activity outcomes to measure;	Vigorous intensity physical activity, time
Physical Activity outcomes to measure;	Moderate-Vigorous intensity physical activity, time
Physical Activity outcomes to measure;	Measure at baseline
Physical Activity outcomes to measure;	Measure at follow-up
Report Physical Activity outcomes as;	mins/day
Report Physical Activity outcomes as;	% of daily waking hours
Report Physical Activity outcomes as;	Report as; mean, standard deviation, and range for participants, Pre and Post -intervention (baseline and follow- up)
Report Physical Activity outcomes as;	Report as; change in mean, and confidence interval, for each outcome

* Modified at ratification meeting from Combined Inclinometer/Accelerometer. Inclinometer not ratified

Core Outcome Set-STandards for Reporting: The COS-STAR Statement Checklist

SECTION/TOPIC	ITEM No.	CHECKLIST ITEM	REPORTED ON PAGE NUMBER
TITLE/ABSTRACT			
Title	1a	Identify in the title that the paper reports the development of a COS	1
Abstract	1b	Provide a structured summary	1
INTRODUCTION			
Background and Objectives	2a	Describe the background and explain the rationale for developing the COS.	1-2
	2b	Describe the specific objectives with reference to developing a COS.	2
Scope	3a	Describe the health condition(s) and population(s) covered by the COS.	2
	3b	Describe the intervention(s) covered by the COS.	2
	3c	Describe the setting(s) in which the COS is to be applied.	2
METHODS			
Protocol/Registry Entry	4	Indicate where the COS development protocol can be accessed, if available, and/or the study registration details.	3
Participants	5	Describe the rationale for stakeholder groups involved in the COS development process, eligibility criteria for participants from each group, and a description of how the individuals involved were identified.	4
Information Sources	6a	Describe the information sources used to identify an initial list of outcomes.	3
	6b	Describe how outcomes were dropped/combined, with reasons (if applicable).	5, 7
Consensus Process	7	Describe how the consensus process was undertaken.	5-7
Outcome Scoring	8	Describe how outcomes were scored and how scores were summarised.	6-7
Consensus Definition	9a	Describe the consensus definition.	5
	9b	Describe the procedure for determining how outcomes were included or excluded from consideration during the consensus process.	6
Ethics and Consent	10	Provide a statement regarding the ethics and consent issues for the study.	12
RESULTS			
Protocol Deviations	11	Describe any changes from the protocol (if applicable), with reasons, and describe what impact these changes have on the results.	n/a
Participants	12	Present data on the number and relevant characteristics of the people involved at all stages of COS development.	4-5

Outcomes	13a	List all outcomes considered at the start of the consensus process.	Supplementary table S1
	13b	Describe any new outcomes introduced and any outcomes dropped, with reasons, during the consensus process.	Supplementary table S5 and S6
COS	14	List the outcomes in the final COS.	12 and supplementary table S7
DISCUSSION			
Limitations	15	Discuss any limitations in the COS development process.	11
Conclusions	16	Provide an interpretation of the final COS in the context of other evidence, and implications for future research.	8-11
OTHER INFORMATION			
Funding	17	Describe sources of funding/role of funders.	12
Conflicts of Interest	18	Describe any conflicts of interest within the study team and how these were managed.	12

From: Kirkham JJ, Gorst S, Altman DG, Blazeby JM, Clarke M, Devane D, et al. (2016) Core Outcome Set–STAndards for Reporting: The COS-STAR Statement. PLoS Med 13(10): e1002148. https://doi.org/10.1371/journal.pmed.1002148
